# Entropy Production and Filling Time in Hydrogen Refueling Stations: An Economic Assessment

**DOI:** 10.3390/e26090735

**Published:** 2024-08-29

**Authors:** Bruno F. Santoro, David Rincón, Diego F. Mendoza

**Affiliations:** 1 Op2B—Optimization to Business, Av. Pompéia, 723, São Paulo 05023-000, Brazil; bruno.santoro@op2b.com.br; 2 Department of Chemical and Biomolecular Engineering, University of California, Los Angeles, CA 90095, USA; fdrinconc@g.ucla.edu; 3 Department of Chemical Engineering, Universidad de Antioquia, Medellín 050010, Colombia

**Keywords:** dynamic optimization, energy efficiency, vehicle fueling, irreversibility analysis

## Abstract

A multi-objective optimization is performed to obtain fueling conditions in hydrogen stations leading to improved filling times and thermodynamic efficiency (entropy production) of the de facto standard of operation, which is defined by the protocol SAE J2601. After finding the Pareto frontier between filling time and total entropy production, it was found that SAE J2601 is suboptimal in terms of these process variables. Specifically, reductions of filling time from 47 to 77% are possible in the analyzed range of ambient temperatures (from 10 to 40 °C) with higher saving potential the hotter the weather conditions. Maximum entropy production savings with respect to SAE J2601 (7% for 10 °C, 1% for 40 °C) demand a longer filling time that increases with ambient temperature (264% for 10 °C, 350% for 40 °C). Considering average electricity prices in California, USA, the operating cost of the filling process can be reduced between 8 and 28% without increasing the expected filling time.

## 1. Introduction

Transportation using hydrogen fuel cells has brought new research opportunities for sustainable energy sources for personal and mass transit systems. For example, the Cal State LA Hydrogen Research and Fueling Facility has pioneered hydrogen on-site production for vehicles using electrolysis since 2014 [1]. The University of São Paulo projects for 2024 include a fueling station for renewable hydrogen from ethanol for buses and vehicles [2]. Similarly, Hynion and Toyota Norway have agreed to cooperate in expanding the hydrogen refueling station at Høvik [3]. To be economically competitive with traditional systems, fueling facilities for hydrogen transportation must be optimized from the energy expenditure point of view and user experience, which is affected among other factors by the filling time.

Hydrogen refueling stations (HRSs) technology has promised similar filling time as what is achieved with usual combustion engine vehicles. To attain such a goal, protocol SAE J2601 has been embedded in the HRSs system. In general lines, it simply specifies a pressure ramp to be provided to the system, whose slope is a function of the initial state of charge of the vehicle tank and the atmospheric temperature [4]. In addition, the SAE J2601 design is based only on the first law of thermodynamics. In order to avoid the static approach of SAE J2601 and improve filling time, first-law approaches have been proposed for HRSs using optimal control [5,6]. Still, entropy production for HRSs has been explored only recently, when minimal entropy production paths were identified to outperform SAE J2601 with 20 to 27% in energy savings, for a fixed filling time of 400 s for both approaches [7]. Due to space constraints, more details about SAE J2601 and other first-law contributions in the literature of HRSs are found elsewhere [7].

For other applications, there is a vast literature considering the second law of thermodynamics in process design and operation. By using a combination of entropy production minimization and nature-inspired designs, it was found that cylinder-shaped tubular reactors have less uniform entropy production than reactors with varying radius along the tube [8]. Energy (i.e., 50%) and cooling (i.e., 30%) savings were found when implementing entropy production minimization in an adiabatic distillation column for a binary system using rate-based and equilibrium-stage models [9]. Multiple entropy production minimization methods such as the equipartition of forces, equipartition of entropy production, and equal thermodynamic speed were compared in the same system in order to understand the effect of their assumptions on the optimal solution [10]. In heat transfer processes, a constant local entropy production rate showed lower entropy production than equipartition of thermal driving force [11]. Many other studies on entropy production have shown the benefits of this approach when optimizing classical chemical processes [12,13,14,15].

In this contribution, the filling time of HRSs is optimized by using an entropy balance together with a validated first-principles model. The proposed approach is compared with the protocol SAE J2601 under different scenarios of ambient temperatures. A multi-objective optimization is performed to obtain fueling conditions leading to optimal filling times with maximal thermodynamic efficiency quantified by the entropy production. As a result, a utopia-tracking approach representation is presented in terms of a Pareto frontier. Finally, the operating cost per filling cycle (i.e., economic analysis) of HRSs under the proposed approach and protocol SAE J2601 are compared under different scenarios.

There are three main contributions of this work:

- To include entropy production in a high-fidelity thermodynamic model of a hydrogen refueling station.
- To minimize the filling time of the HRS system under entropy production using optimal control and to compare it with SAE J2601 in a multi-objective approach.
- To analyze the operating cost of the HRS for different operating conditions.

## 2. Materials and Methods

### 2.1. Station Layout

**Figure 1:**
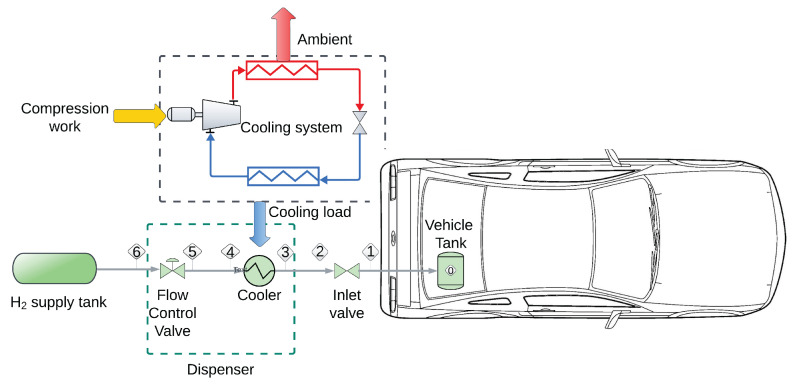
Hydrogen station layout.

A simplified version of an HRS is shown in Figure 1. The refueling process starts when hydrogen is dispensed from the high-pressure supply tanks (stream 6) with the hydrogen flow regulated by the flow control valve. Subsequently, the hydrogen exiting the control valve (stream 5) is thermally conditioned in a cooler that supplies the required cooling load through a refrigeration system. Once the hydrogen flow rate and temperature are adequate, hydrogen enters the vehicle tank through the inlet valve.

### 2.2. Mathematical Model

Olmos and Manousiouthakis [5] presented a mathematical model for an HRS similar to the one presented in Figure 1, using mass and energy balances for each component in the system (i.e., tank, cooler, etc.). Later, such a model was expanded by Mendoza et al. [7] by including a high-accuracy thermodynamic model, valve pressure drop equations, and entropy balances for the main components of the system. In the mathematical representation of the refueling station, it is assumed that the dynamics of the cooler and valves are significantly faster than those of the tank. Consequently, the valves and cooler dynamics are represented by a set of algebraic equations, while the tank is modeled using differential equations.

In the current proposal, mass and energy balances for the hydrogen in the vehicle tank are given by Equations (1) and (2), respectively.

(1) $$\begin{array}{c} V_{0}\frac{d\rho_{0}\left(t\right)}{dt}=\dot{m}\left(t\right) \end{array}$$

where $V_{0}$ is the vehicle tank capacity, $\rho_{0}$ is the density of the hydrogen confined in the tank, and $\dot{m}$ is the hydrogen mass flow rate entering the tank. The left-hand side of the mass balance, Equation (1), represents the accumulation rate of hydrogen inside the tank due to the hydrogen supplied through the inlet valve, iv.

The energy balance for the gas confined in the vehicle tank is

(2) $$V_{0}\rho_{0}\left(t\right)\frac{du_{0}\left(t\right)}{dt}=\dot{m}\left(t\right)\left[u_{1}\left(t\right)-u_{0}\left(t\right)+\frac{p_{1}\left(t\right)}{\rho_{1}\left(t\right)}\right]-{\dot{Q}}_{w0}\left(t\right)$$

where $u_{0}$, $u_{1}$ represent the internal energy of hydrogen in the tank and in stream 1, respectively; $p_{1}$, $\rho_{1}$ are the pressure and density of hydrogen at conditions of stream 1, and ${\dot{Q}}_{w0}$ is the heat transfer rate between the gas and the vehicle tank wall. The left-hand side of Equation (2) denotes the internal energy accumulation rate due to the energy interactions with the wall and with the hydrogen entering the tank.

The tank-wall dynamics is described by an energy balance, Equation (3), where the rate of change of the wall temperature, $T_{w}$, is a consequence of the heat interactions with the hydrogen in the tank, ${\dot{Q}}_{wo}$, and with the environment, ${\dot{Q}}_{w\infty }$. These heat flow rates are calculated using Newton’s cooling law, which is represented by Equations (4) and (5), respectively.

(3) $$\begin{array}{cc} \rho_{w}V_{w}Cp_{w}\frac{dT_{w}\left(t\right)}{dt} & ={\dot{Q}}_{w0}\left(t\right)-{\dot{Q}}_{w\infty }\left(t\right) \end{array}$$

(4) $$\begin{array}{cc} {\dot{Q}}_{w0}\left(t\right) & =h_{w0}A_{w0}\left[T_{0}\left(t\right)-T_{w}\left(t\right)\right] \end{array}$$

(5) $$\begin{array}{cc} {\dot{Q}}_{w\infty }\left(t\right) & =h_{w\infty }A_{w\infty }\left[T_{w}\left(t\right)-T_{\infty }\left(t\right)\right] \end{array}$$

where, $\rho_{w}$, $V_{w}$, $Cp_{w}$ are the density, volume and heat capacity of the vehicle tank wall, respectively. $h_{w0}$, $h_{w\infty }$ are the hydrogen/wall and wall/air convective heat transfer coefficients; $A_{w0}$, $A_{w\infty }$ are the heat transfer areas, and $T_{\infty }$ is the temperature of the environment.

The cooling system is described by the energy balance in the cooler, Equation (6), and by the coefficient of performance (COP) of the refrigeration unit as defined in Equation (7).

(6) $$\begin{array}{cc} u_{4}\left(t\right)+\frac{p_{4}\left(t\right)}{\rho_{4}\left(t\right)}+q_{c}\left(t\right) & =u_{3}\left(t\right)+\frac{p_{3}\left(t\right)}{\rho_{3}\left(t\right)} \end{array}$$

(7) $$\begin{array}{cc} COP(t) & =\frac{w_{c}\left(t\right)}{q_{c}\left(t\right)} \end{array}$$

where $w_{c}$ is the compression work input in the cooling unit and $q_{c}$ is the heat transfer duty in the cooler. The variation of the COP as a function of the ambient temperature [16] is given by Equation (8).

(8) $$COP\left(t\right)=1.6\exp(-0.018\left(T_{\infty }\left(t\right)-273.15\right))$$

The inlet valve, iv, is modeled using an energy balance, Equation (9), and a design equation, Equation (10). Similarly, the flow control valve, fv, is modeled using an energy balance and a design equation, which are given by Equations (11) and (12), respectively.

(9) $$\begin{array}{cc} u_{2}\left(t\right)+\frac{p_{2}\left(t\right)}{\rho_{2}\left(t\right)} & =u_{1}\left(t\right)+\frac{p_{1}\left(t\right)}{\rho_{1}\left(t\right)} \end{array}$$

(10) $$\begin{array}{cc} \frac{\dot{m}\left(t\right)}{\rho_{N}} & =\frac{514}{3.6\times 10^{5}}k_{v,iv}\sqrt{\frac{p_{1}\left(t\right)\left(p_{2}\left(t\right)-p_{1}\left(t\right)\right)}{\rho_{N}T_{2}\left(t\right)}} \end{array}$$

(11) $$\begin{array}{cc} u_{6}\left(t\right)+\frac{p_{6}\left(t\right)}{\rho_{6}\left(t\right)} & =u_{5}\left(t\right)+\frac{p_{5}\left(t\right)}{\rho_{5}\left(t\right)} \end{array}$$

(12) $$\begin{array}{cc} \frac{\dot{m}\left(t\right)}{\rho_{N}} & =\frac{514}{3.6\times 10^{5}}k_{v,fv}\left(t\right)\sqrt{\frac{p_{5}\left(t\right)\left(p_{6}-p_{5}\left(t\right)\right)}{\rho_{N}T_{5}\left(t\right)}} \end{array}$$

where $k_{v}$ is the valve coefficient and $\rho_{N}$ is the density at normal conditions (273.15 K and 101.325 kPa). The main difference between the valves is that the inlet valve is assumed to be completely open during the filling process, while the opening fraction of the flow control may be changed, $0\leq x_{fv}\left(t\right)\leq 1$. A detailed description of the variables and its units is provided in the Abbreviations section.

### 2.3. Entropy Production

The total entropy production at the end of the fueling process, $S_{irr}\left(t_{f}\right)$, is calculated from the overall entropy balances for each component of the station,

(13) $$S_{irr}\left(t_{f}\right)=\sum_{k\in K}\int_{0}^{t_{f}}{\left(\frac{dS(t)}{dt}\right)}_{irr,k}dt,\;K\in \left\{0,w,iv,fv\right\}$$

where ${\left(dS/dt\right)}_{irr,k}$ is the instantaneous entropy production rate in component *k*, namely: vehicle tank (0), tank wall *w*, inlet valve (iv) and flow control valve (fv).

**Remark 1.**  *Flow in pipes is assumed to be adiabatic and reversible, and heat transfer in the cooler is assumed to be reversible; both assumptions are necessary simplifications due to the lack of details about the actual pipework and heat exchanger type and flow configuration. It is important to notice that in *(13)*, the final fueling time $t_{f}$ could be replaced by a particular time instant t to obtain the cumulative entropy generation up to that time.*

The entropy production in the gas confined in the tank, Equation (14), is calculated as the difference between the entropy outlet and inlet flows. The first term on the right-hand side corresponds to the outlet entropy flow due to the heat interaction with the tank wall, while the second and third terms correspond to the entropy flow due to the inlet hydrogen flow and to the instantaneous accumulation rate of entropy by the mass confined in the tank, respectively.

(14) $${\left(\frac{dS(t)}{dt}\right)}_{irr,0}=\frac{{\dot{Q}}_{w0}\left(t\right)}{T_{w}\left(t\right)}-\dot{m}\left(t\right)\;s_{1}\left(t\right)+V_{0}\frac{d\rho_{0}\left(t\right)s_{0}\left(t\right)}{dt}$$

The term $d\rho_{0}\left(t\right)s_{0}\left(t\right)/dt$ in the in the preceding equation is expanded as follows:

(15) $$V_{0}\frac{d\rho_{0}\left(t\right)s_{0}\left(t\right)}{dt}=V_{0}\rho_{0}\left(t\right)\frac{ds_{0}\left(t\right)}{dt}+s_{0}\left(t\right)V_{0}\frac{d\rho_{0}\left(t\right)}{dt}$$

where $ds_{0}\left(t\right)/dt$ is calculated from the fundamental equation of thermodyamics for a simple compressible substance,

(16) $$\frac{ds_{0}\left(t\right)}{dt}=\frac{1}{T_{0}\left(t\right)}\frac{du_{0}\left(t\right)}{dt}-\frac{p_{0}\left(t\right)}{T_{0}\left(t\right)\rho_{0}^{2}\left(t\right)}\frac{d\rho_{0}\left(t\right)}{dt}$$

combining Equations (1), (2), (15) and (16) into Equation (14) yields the following expression

(17) $$\begin{array}{cc} {\left(\frac{dS(t)}{dt}\right)}_{irr,0}= & {\dot{Q}}_{w0}\left(t\right)\left(\frac{1}{T_{w}\left(t\right)}-\frac{1}{T_{0}\left(t\right)}\right)\\ & +\dot{m}\left(t\right)\left(u_{1}\left(t\right)+\frac{p_{1}\left(t\right)}{\rho_{1}\left(t\right)}\right)\left(\frac{1}{T_{0}\left(t\right)}-\frac{1}{T_{1}\left(t\right)}\right)+\dot{m}\left(t\right)\left(\frac{\mu_{1}\left(t\right)}{T_{1}\left(t\right)}-\frac{\mu_{0}\left(t\right)}{T_{0}\left(t\right)}\right) \end{array}$$

The terms on the right-hand side of Equation (17) show the entropy production as the sum of flows and thermodynamic forces related to heat transfer between the hydrogen confined in the tank and the wall (first term), with the temperature difference between the inlet gas and the hydrogen in the tank (second term), and with the chemical potential, μ, difference between the hydrogen entering the tank and the hydrogen confined in the tank (third term).

Entropy production within the wall arises from thermal interactions with both the confined gas and the environment,

(18) $${\left(\frac{dS(t)}{dt}\right)}_{irr,w}=\frac{{\dot{Q}}_{w\infty }\left(t\right)}{T_{w\infty }}-\frac{{\dot{Q}}_{w0}\left(t\right)}{T_{w}\left(t\right)}+\frac{\rho_{w}V_{w}Cp_{w}}{T_{w}\left(t\right)}\;\frac{dT_{w}\left(T\right)}{dt}$$

The last term on the right-hand side of Equation (18) accounts for the entropy accumulation rate in the wall. Substituting the last term on the right-hand side of Equation (18) by the energy balance on the wall, Equation (3), we obtain the entropy production in the wall, Equation (19), as a product of the heat flow and the heat transfer thermodynamic force, i.e., the temperature difference between the wall and the environment.

(19) $${\left(\frac{dS(t)}{dt}\right)}_{irr,w}={\dot{Q}}_{w\infty }\left(t\right)\left(\frac{1}{T_{\infty }}-\frac{1}{T_{w}\left(t\right)}\right)$$

Entropy production within the adiabatic valves is attributed to the difference between entropy flows carried by the outlet and inlet mass streams.

(20) $${\left(\frac{dS(t)}{dt}\right)}_{irr,iv}=\dot{m}\left(t\right)s_{1}\left(t\right)-\dot{m}\left(t\right)s_{2}\left(t\right)$$

(21) $${\left(\frac{dS(t)}{dt}\right)}_{irr,fv}=\dot{m}\left(t\right)s_{5}\left(t\right)-\dot{m}\left(t\right)s_{6}\left(t\right)$$

An insight about the nature of the irreversibility associated with the expansion in the adiabatic valve can be obtained using differential expressions for the entropy and enthalpy in the valves, as shown in Equations (22) and (23), respectively.

(22) $$ds=-\frac{\partial }{\partial T}{\left(\frac{1}{\rho }\right)}_{p}dp+\frac{C_{p}}{T}dT$$

(23) $$dh=\left[\frac{1}{\rho }-T\frac{\partial }{\partial T}{\left(\frac{1}{\rho }\right)}_{p}\right]+C_{p}dT=0$$

The constant pressure heat capacity, $C_{p}$, obtained from Equation (23) is replaced in Equation (22) providing an expression for the entropy change in the adiabatic valve as

(24) $$ds=-\frac{dp}{\rho T}$$

From this expression, we obtain the entropy production in the valves

(25) $${\left(\frac{dS(t)}{dt}\right)}_{irr,iv}=-\dot{m}\left(t\right)\int_{p_{2}\left(t\right)}^{p_{1}\left(t\right)}\frac{dp}{\rho (t)T(t)}$$

(26) $${\left(\frac{dS(t)}{dt}\right)}_{irr,fv}=-\dot{m}\left(t\right)\int_{p_{6}\left(t\right)}^{p_{5}\left(t\right)}\frac{dp}{\rho (t)T(t)}$$

Equations (25) and (26) show that the entropy production in the valves is proportional to the instantaneous pressure drop in them.

### 2.4. Thermophysical Properties

The thermophysical and geometry parameters for the vehicle tank presented in Table 1 were obtained from the work of Olmos and Manousiouthakis [5].

All the thermodynamic properties of hydrogen were determined from a fundamental Helmholtz equation [17]. Detailed expressions for these properties are provided in Appendix A.

### 2.5. Optimization Problem

The minimization of filling time corresponds to an optimal control problem, where the inlet flow rate and the cooling load can be manipulated to achieve the fastest refueling while satisfying process safety constraints. Before posing an optimization problem for such a goal, it is necessary to manipulate the differential equations of the model to allow the simulation horizon to be taken as a variable. This can be accomplished through a scaling factor $t_{f}$, which is a decision variable of the problem corresponding to the final time of filling. Then, let $\tau =t/t_{f}$ be a dimensionless time to replace *t* in the model equations. From its definition, it is clear that the domain of integration is between 0 and 1, i.e., 0≤τ≤1. With this consideration, Equation (1) is rewritten as

(27) $$V_{0}\frac{1}{t_{f}}\frac{d\rho_{0}\left(\tau \right)}{d\tau }=\dot{m}\left(\tau \right)$$

After this change in variables in all equations, the optimization problem is

(28) $$\begin{array}{cc} \; & \min\int_{0}^{t_{f}}1\;dt=t_{f} \end{array}$$

$$\begin{array}{cc} \; & s.t.\\ \; & \mathrm{Equations}\;(1)\;\mathrm{to}\;(12) \end{array}$$

(29) $$\begin{array}{cc} \; & 0\leq \dot{m}\left(\tau \right)\leq {\dot{m}}_{max}\; \end{array}$$

(30) $$\begin{array}{cc} \; & 0\leq x_{fv}\left(\tau \right)\leq 1\; \end{array}$$

(31) $$\begin{array}{cc} \; & q_{c,max}\leq q_{c}\left(\tau \right)\leq 0\; \end{array}$$

(32) $$\begin{array}{cc} \; & SOC\left(\tau =1\right)\geq SOC_{des}\; \end{array}$$

(33) $$\begin{array}{cc} \; & S_{irr}\left(\tau =1\right)\leq S_{irr}^{max}\; \end{array}$$

Equations (29)–(31) are bounds on the manipulated variables, accounting for the physical limitations of the filling station. The constraint imposed by Equation (32) forces the state of charge (equivalent to the total amount of hydrogen in the tank) at the end of simulation horizon to achieve the desired value, $SOC_{des}$. In the results outlined in Section 3, the dynamic profile of all variables is converted back from τ to the dimensional value *t* for easier visualization and comparison with other techniques in the literature.

Entropy production is a secondary objective of the formulation. To deal with this multi-objective situation, Equation (33), an upper bound on the total generated entropy, is used in an ε-constraint method. More explicitly, $S_{irr}^{max}$ is initially set as a sufficiently large value in order for the constraint defined by Equation (33) to be inactive, allowing the calculation of the absolute minimum filling time. Progressively, lower values of $S_{irr}^{max}$ are chosen and lead to new points in the Pareto frontier (PF) of optimal solutions with minimal time and entropy production.

### 2.6. Economic Assessment

The model presented in Section 2.2 allows the calculation of energy consumption per filling cycle. In order to convert this expenditure into economic values, the commercial electric rates of Irvine, California (USA) in 2023 were considered as a benchmark [18]. In many regions, it is common for energy prices to change accordingly to the seasons of the year or even during a single day. In this study, only the monthly variation was considered by taking three data points: the highest (0.276 $/kWh, in July), the lowest (0.186 $/kWh, in February) and a typical value (0.204 $/kWh, in November).

### 2.7. Optimization Implementation and Solution Method

The problem was formulated using Pyomo [19], in particular with pyomo.dae [20] as a tool to convert the differential equations into an algebraic equivalent. Discretization has been made with 30 finite elements and two Radau collocation points per element. The resulting non-linear program was solved using CONOPT4. The typical computational time is around 20–30 s per simulation in a Windows 11 environment, using an Intel^®^ Core™ i7-11800 CPU 2.30 GHz processor and 32 GB of RAM memory. The implemented model was validated in comparison with experimental data, achieving maximum deviations of around 1% in terms of state of charge and 7% of hydrogen temperature. Further details on model validation are provided in Ref. [7].

## 3. Results

### 3.1. Filling Time and Entropy Production

In order to evaluate the trade-off between filling time and thermodynamic efficiency, several simulations were conducted considering different ambient temperatures: 10, 25 and 40 °C. The simulations in this work consider that the vehicle tank is of type IV, starting at 5 MPa and the same temperature as the ambient. The filling station is considered to be H70-T40 (dispenser at 70 MPa and −40 °C), and the end goal is a state of charge of 99.1%.

**Figure 2:**
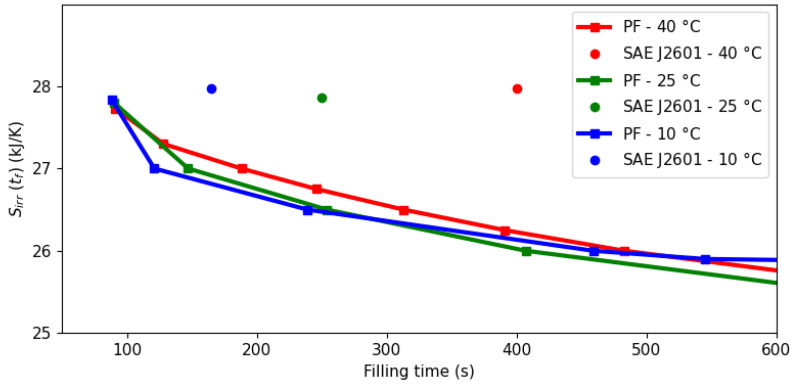
Pareto frontier of the total entropy production in the filling process, $S_{irr}\left(t_{f}\right)$, for an allowed filling time at different ambient temperatures.

A comparison of the optimization approach with the benchmark protocol SAE J2601 is shown in Figure 2. The squares correspond to specific optimizations after choosing particular values for $S_{irr}^{max}$, while the continuous lines joining them are a piece-wise linear approximation of the underlying Pareto frontier.

The pressure ramp specified by SAE J2601 indirectly defines the corresponding filling time given the initial and final pressures. However, Figure 2 shows that such an operation (circular points) lies outside the Pareto frontier and is therefore suboptimal in terms of entropy minimization and filling time. For instance, when the ambient temperature is 40 °C, the protocol takes 400 s and generates 28 kJ/K, while the optimal profile achieves the same state of charge in only 91 s (77% lower) with an entropy production of 27.7 kJ/K (1% lower). The effect is more manifested the greater the ambient temperature, with possible time reductions of 160 s (64%) and 77 s (47%) at 25 °C and 10 °C, respectively.

Minimal entropy production can be achieved with a system operation approaching the thermodynamic limit of reversibility, which implies a slower process and smaller flow rate. Figure 2 shows that to move from the protocol toward a condition of minimal entropy has a significant cost in terms of filling time. For instance, when the ambient temperature is 10 °C, it would be necessary to spend around 600 s (264% increase) to achieve an entropy reduction of only 2 kJ/K (7%). For the 40 °C scenario, the minimum entropy is not shown in Figure 2 for scaling reasons, but it would take around 2000 s (350%) to reduce the entropy production by 3.5 kJ/K (12%). However, small decreases in entropy production may correspond to significant savings in operating costs due to there being smaller work loads in the cooling system.

**Figure 3:**
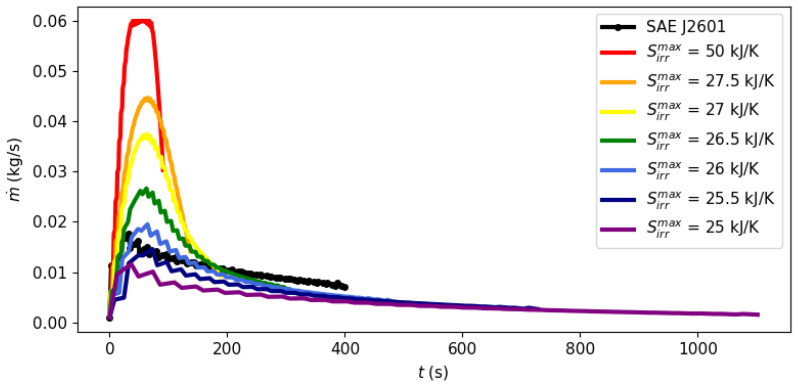
Evolution of the filling flow rate during the refueling process at different points of the Pareto frontier.

**Figure 4:**
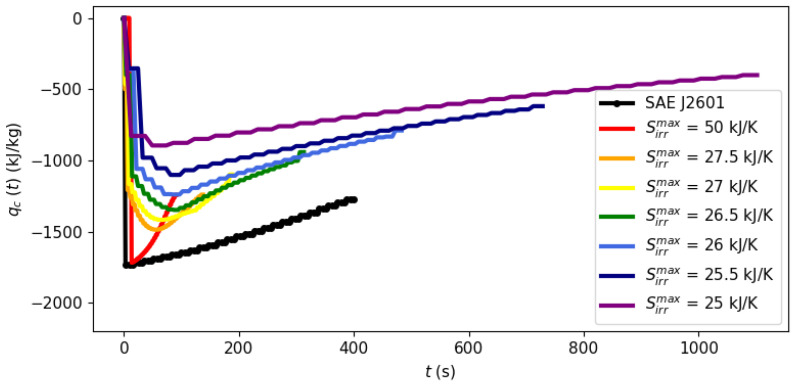
Evolution of the cooling load during the refueling process at different points of the Pareto frontier.

**Figure 5:**
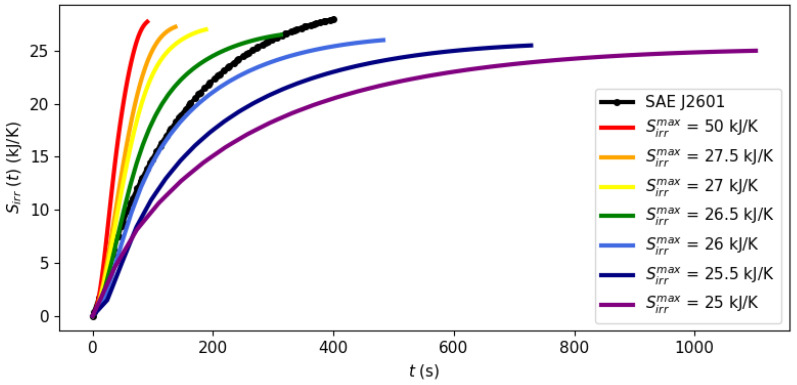
Evolution of the cumulative generated entropy up to time t, $S_{irr}\left(t\right)$, at different points of the Pareto frontier.

System dynamics during the filling process can be observed at different points of the Pareto frontier in Figure 3, Figure 4 and Figure 5 with a fixed ambient temperature of 40 °C. The evolution of the manipulated variables of the control system, flow rate and the cooling load is given in Figure 3 and Figure 4, respectively, while Figure 5 shows the evolution of entropy production.

Figure 3, Figure 4 and Figure 5 illustrate the optimal filling profile at different points of the Pareto frontier. For instance, when time is minimized with a virtually unbounded entropy production ($S_{irr}^{max}$= 50 kJ/K, red curves), the flow rate quickly achieves its maximum allowed value of ${\dot{m}}_{max}$ = 0.06 kg/s. Accordingly, the cooling load is more intense when there is a higher flow rate ($S_{irr}^{max}$= 50 kJ/K) or when there is a stricter requirement of inlet temperature (Protocol SAE J2601). As the requirement of maximal entropy production becomes more stringent, it is no longer feasible to operate at a maximum flow rate, dispersing the filling operation over time. Figure 5 shows that an excessively long filling time is necessary for the system to achieve its practical minimal point of entropy production at 25 kJ/K.

**Remark 2.**  *Figure 3, Figure 4 and Figure 5 illustrate a dynamic analysis of the system, meaning that the x-axis corresponds to time as the independent variable of the DAE system, as shown in Equations *(1)*–*(12)*. On the other hand, Figure 2 is a static overview, because its x-axis, “Filling time”, corresponds to $t_{f}$ in the model formulation, i.e., the final time value. In other words, the static information of Figure 2 is contained in the dynamic plot of Figure 5, which shows the cumulative entropy generation up to each possible time t, including $t_{f}$. Notice that the end points of the entropy production trajectories in Figure 5 are exactly the Pareto frontier for the system at 40 °C that was presented in Figure 2.*

### 3.2. Economic Analysis

**Figure 6:**
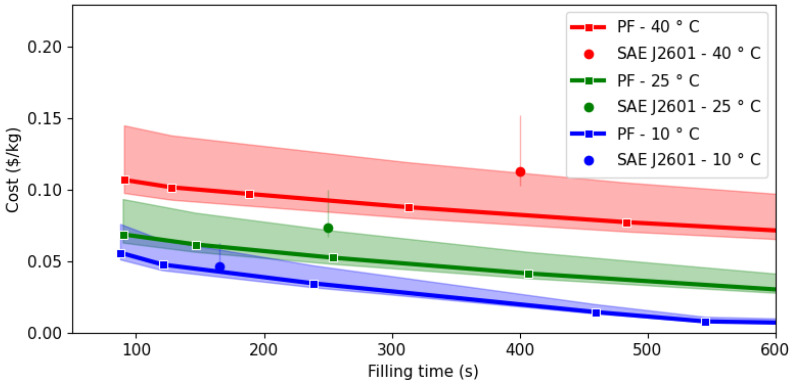
Pareto frontier for cost as a function of ambient temperature.

The trade-off between operating costs and filling time has been analyzed for three ambient temperatures (10, 25 and 40 °C) and typical seasonal electricity costs in Irvine (California, USA), in the Los Angeles metropolitan area. Results are shown in Figure 6, with the continuous line corresponding to usual electricity rates, while the shadowed zone represents the range between minimum and maximum values registered in 2023. The circles correspond to costs associated with filling according to the protocol SAE J2601, which once again lie outside the Pareto frontier.

Given that the vehicle tanks considered in this study have a capacity of 4.8 kg, to fill them from an initial pressure of 5 MPa and at ambient temperature to an SOC of 99.1% corresponds to a total mass of 4.3 kg to be refueled. Therefore, the total operational costs associated with a refueling of this magnitude when using the protocol SAE J2601 range from $0.19 (best-case scenario, with 10 °C ambient temperature and lowest electricity rate) to $0.66 (40 °C, highest electricity rate) per filling.

The order of magnitude of the operational cost agrees with the ones reported in other studies. For instance, [21] analyzed costs of HRSs in Germany and found compressor costs between 0.08 and 0.18 €/kg. In comparison with total hydrogen prices, ref. [22] placed a value around 10 €/kg considering the whole supply chain, which indicates that the bottleneck still lies in how to obtain the fuel in a cost-effective manner.

Ambient temperature plays a relevant role in the operational costs, since these increase with temperature, either using protocol SAE J2601 or the thermodynamic optimized filling process. This observation agrees with the fact that higher ambient temperatures demand more work from the cooling system regardless of the filling process followed, as shown in Table 2. However, the thermodynamic optimized process outperforms the SAE J2601 in all assessed scenarios, such that for the same filling time, the operation costs could be reduced by 8% at 10 °C, 28% at 25 °C, and 27% at 40 °C with respect to the SAE J2601 protocol.

In contrast to SAE J2601, the thermodynamic optimized operation allows the possibility of devising flexible operation with variable fueling times along the Pareto frontier. One case of interest would be having fast refueling with the same operation cost as SAE J2601: under this scenario, the filling times could be reduced between 40 s (10 °C) and 300 s (40 °C), allowing the station to achieve a higher throughput during the day when required (e.g., peak hours).

Considering a time delay of two minutes between vehicles and opening hours for the HRS between 8 AM and 8 PM, then it is possible to service between 83 (with outside temperature equal to 40 °C) and 151 vehicles (at 10 °C) if the SAE J2601 filling protocol is applied. If the operating costs are kept at the same level, the filling time could be reduced to around 90 s for temperatures above 10 °C, which in turn would allow around 200 vehicles to be serviced (increase between 54 and 120%).

On the other hand, if more emphasis is put on reducing costs while keeping the filling time of the protocol, it is possible to achieve higher savings. For instance, if a filling time equal to 500 s is considered acceptable for all temperatures, which is less than 10% slower than the SAE J2601 protocol for 40 °C, then savings of around 0.036 $/kg are possible for all temperatures. If 100 vehicles are serviced every day, the total savings amount to more than $5500 per HRS, which would compensate the additional investment required to operate under an optimal controller.

## 4. Conclusions

This study has evaluated the interdependency among filling time, entropy production and operating cost in HRSs. It highlights that the de facto standard of operation, defined by the protocol SAE J2601, is suboptimal in terms of these process variables, because it lies outside the Pareto frontier. Reductions of filling time from 47 to 77% are possible in the analyzed range of ambient temperature (from 10 to 40 °C) with higher savings potential the hotter the weather conditions.

Entropy production savings seem to demand a disproportionate increase in filling time; however, the economic analysis reveals that operating costs may be reduced by 30% with filling times 10% higher than what is obtained with the SAE J2601 protocol at 40 °C, which could be possible during off-peak hours.

Finally, the operating cost analysis shows possibilities for flexible operations to generate price policies which could be adapted to either fast, slow and normal fueling times with respect to the SAE J2601 protocol; this flexibility could facilitate the public adoption of hydrogen in the transportation sector, since both price and filling time can be adapted to customer needs. The identified economic and energy improvements provide a basis for developing more detailed mathematical models, particularly for the cooling system. A deeper economic analysis can subsequently enable a refined thermo-economic optimization of the refueling protocol. Further work could be conducted to enhance the safety aspects of SAE2601 by using constraint-based methodologies and/or barrier functions.

## Figures and Tables

**Table 1:** Vehicle tank wall properties [5].

Thermophysical		Geometry	
$h_{wi}$ (kW m^−2^ K^−1^)	0.06	$V_{0}$ (m^3^)	0.12
$h_{w0}$ (kW m^−2^ K^−1^)	0.006	$V_{w}$ (m^3^)	0.0576
$\rho_{w}$ (kg m^−3^)	1550	$A_{wi}$ (m^2^)	2.34
$c_{w}$ (kJ kg^−1^ K^−1^)	1.374	$A_{wi}$ (m^2^)	2.88

**Table 2:** Compression work at different ambient temperatures.

Ambient Temperature (°C)	Compression Work (kJ)		
	SAE J2601	Min. time ($S_{irr}^{max}\left(t_{f}\right)$= 50 kJ/K)	Min. time ($S_{irr}^{max}\left(t_{f}\right)$= 26 kJ/K)
10 °C	3572 kJ	3225 kJ	748 kJ
25 °C	5664 kJ	5277 kJ	3117 kJ
40 °C	8625 kJ	8185 kJ	5930 kJ

## Data Availability

Dataset available on request from the authors.

## Abbreviations

The following abbreviations are used in this manuscript:

*Latin*	
*A*	Area, m^2^
*a*	Helmholtz equation parameter, -
*b*	Helmholtz equation parameter, -
*D*	Helmholtz equation parameter, -
DAE	Differential algebraic equations
K	Index
*k*	Valve flow capacity coefficient, m^3^ h^−1^
*h*	Heat transfer coefficient, kW m^−2^ K^−1^
HRS	Hydrogen refueling station
$\dot{m}\left(t\right)$	Mass flow rate, kg s^−1^
p(t)	Pressure, kPa
$p_{c}$	Critical pressure, kPa
PF	Pareto frontier
ps	Pipe segment, -
q(t)	Specific heat flow, kJ kg^−1^
*R*	Ideal gas constant, kJ kg^−1^ K^−1^
S(t)	Entropy, kJ K^−1^
$S_{irr}\left(t\right)$	Total entropy production, kJ K^−1^
s(t)	Specific entropy, kJ kg^−1^ K^−1^
SOC	State of charge
T(t)	Temperature, K
*t*	Time, s
$t_{i}$	Helmholtz equation parameter, -
$T_{c}$	Critical temperature, K
u(t)	Specific internal energy, kJ kg^−1^
*V*	Vehicle tank volume, m^3^

**Subscripts**	
*Greek*	
α	Helmholtz equation parameter, -
δ	Helmholtz equation parameter, -
γ	Helmholtz equation parameter, -
ϕ	Helmholtz equation parameter, -
ρ(t)	Density, kg m^−3^
$\rho_{N}$	Density at normal conditions (273.15 K, 101.325 kPa), kg m^−3^
$\rho_{c}$	Critical density, kg m^−3^
$\rho_{full}$	Maximum admissible density in the vehicle tank kg m^−3^
τ	Helmholtz equation parameter, -
*f*	Final
fv	Flow control valve
*i*	Index
iv	Inlet valve
irr	Irreversible
*k*	Index
*w*	Wall
0	Tank
32	Pipe segment 32
54	Pipe segment 54
*∞*	Ambient
0	Ideal gas
*r*	Residual
ref	Reference state
*k*	Index

## Appendix A. Thermodynamic Properties of Hydrogen

Leachman and coworkers [17] proposed a Helmholtz equation to describe the thermodynamic properties of hydrogen of the form

(A1) $$\frac{a}{RT}=\alpha^{0}\left(\tau ,\delta \right)+\alpha^{r}\left(\tau ,\delta \right)$$

where $\alpha^{0}$ and $\alpha^{r}$ are the ideal gas and residual contributions to the dimensionless Helmholtz energy; both are functions of the reduced density δ = $\rho /\rho_{c}$ and the inverse reduced temperature τ = $T_{c}/T$.

(A2) $$\alpha^{0}\left(\tau ,\delta \right)=\ln\delta +1.5\ln\tau +a_{1}+a_{2}\tau +\sum_{k=3}^{7}a_{k}\ln\left[1-\exp\left(b_{k}\tau \right)\right]$$

(A3) $$\begin{array}{c} \alpha^{r}\left(\tau ,\delta \right)=\sum_{i=1}^{7}N_{i}\delta^{d_{i}}\tau^{t_{i}}+\sum_{i=8}^{9}N_{i}\delta^{d_{i}}\tau^{t_{i}}\exp\left(-\delta^{p_{i}}\right)\\ \;\;\;\;\;\;\;\;\;\;\;\;\;\;+\sum_{i=10}^{14}N_{i}\delta^{d_{i}}\tau^{t_{i}}\exp\left[\phi_{i}{\left(\delta -D_{i}\right)}^{2}+\beta_{i}{\left(\tau -\gamma_{i}\right)}^{2}\right] \end{array}$$

Table A1 and Table A2 provide the values of parameters needed in Equations (A2) and (A3).

From Equation (A1) and its derivatives, any other thermodynamic property of the system is calculated. In these contributions, it suffices to obtain a pressure–density–temperature relation (*p*–ρ–*T*), the internal energy *u*, the entropy *s* and the chemical potential, μ, of the substance.

(A4) $$\frac{p}{\rho RT}=-\frac{\rho }{RT}{\left(\frac{\partial a}{\partial \rho }\right)}_{T}$$

(A5) $$\frac{u}{RT}=\frac{a}{RT}-\frac{1}{R}{\left(\frac{\partial a}{\partial T}\right)}_{\rho }$$

(A6) $$\frac{s}{R}=-\frac{1}{R}{\left(\frac{\partial a}{\partial T}\right)}_{\rho }$$

(A7) $$\frac{\mu }{RT}=\frac{a}{RT}+\frac{p}{\rho RT}$$

The derivatives needed in Equations (A4)–(A6) follow the expressions:

(A8) $$\begin{array}{c} {\left(\frac{\partial \alpha^{r}}{\partial \delta }\right)}_{\tau }=\sum_{i=1}^{7}N_{i}d_{i}\delta^{d_{i}-1}\tau^{t_{i}}+\sum_{i=8}^{9}N_{i}\delta^{d_{i}}\tau^{t_{i}}\exp\left(-\delta^{p_{i}}\right)\left[\frac{d_{i}}{\delta }-p_{i}\frac{\delta^{p_{i}}}{\delta }\right]\\ \;\;\;\;\;\;+\sum_{i=10}^{14}N_{i}\delta_{i}^{d}\tau^{t_{i}}\left[\frac{d_{i}}{\delta }+2\phi_{i}\left(\delta -D_{i}\right)\right]\exp\left[\phi_{i}{\left(\delta -D_{i}\right)}^{2}+\beta_{i}{\left(\tau -\gamma_{i}\right)}^{2}\right] \end{array}$$

(A9) $${\left(\frac{\partial \alpha^{0}}{\partial \tau }\right)}_{\delta }=\frac{1.5}{\tau }+a_{2}-\sum_{k=3}^{7}\frac{a_{k}b_{k}\exp\left(b_{k}\tau \right)}{1-\exp\left(b_{k}\tau \right)}$$

(A10) $$\begin{array}{c} {\left(\frac{\partial \alpha^{r}}{\partial \tau }\right)}_{\delta }=\sum_{i=1}^{7}N_{i}\delta^{d_{i}}t_{i}\tau^{t_{i}-1}+\sum_{i=8}^{9}N_{i}\delta^{d_{i}}t_{i}\tau^{t_{i}-1}\exp\left(-\delta^{p_{i}}\right)\\ \;\;\;\;\;\;+\sum_{i=10}^{14}N_{i}\delta^{d_{i}}\tau^{t_{i}}\left[\frac{t_{i}}{\tau }+2\beta_{i}\left(\tau -\gamma_{i}\right)\right]\exp\left[\phi_{i}{\left(\delta -D_{i}\right)}^{2}+\beta_{i}{\left(\tau -\gamma_{i}\right)}^{2}\right] \end{array}$$

**Table A1:** Coefficients used in $\alpha^{0}$.

*k*	$a_{k}$	$b_{k}$
1	−1.4579856475	-
2	1.888076782	-
3	1.616	−16.0205159149
4	−0.4117	−22.6580178006
5	−0.792	−60.0090511389
6	0.758	−74.9434303817
7	1.217	−206.9392065168

**Table A2:** Coefficients used in $\alpha^{r}$.

*i*	$N_{i}$	$t_{i}$	$d_{i}$	$p_{i}$	$\phi_{i}$	$\beta_{i}$	$\gamma_{i}$	$D_{i}$
1	−6.93643	0.6844	1	-	-	-	-	-
2	0.01	1	4	-	-	-	-	-
3	2.1101	0.989	1	-	-	-	-	-
4	4.52059	0.489	1	-	-	-	-	-
5	0.732564	0.803	2	-	-	-	-	-
6	−1.34086	1.1444	2	-	-	-	-	-
7	0.130985	1.409	3	-	-	-	-	-
8	−0.777414	1.754	1	1	-	-	-	-
9	0.351944	1.311	3	1	-	-	-	-
10	−0.0211716	4.187	2	-	−1.685	−0.1710	0.7164	1.506
11	0.0226312	5.646	1	-	−0.489	−0.2245	1.3444	0.156
12	0.032187	0.791	3	-	−0.103	−0.1304	1.4517	1.736
13	−0.0231752	7.249	1	-	−2.506	−0.2785	0.7204	0.67
14	0.0557346	2.986	1	-	−1.607	−0.3967	1.5445	1.662
